# Full-length 16S rRNA sequencing reveals microbial characteristics in supragingival plaque of periodontitis patients

**DOI:** 10.1016/j.jds.2025.03.040

**Published:** 2025-04-12

**Authors:** Chih-Chieh Wen, Jerry C.Y. Lin, Eisner Salamanca, Nai-Chia Teng, Yi-Jen Lin, Yi-Fan Wu, Hung-Ming Chang, Wei-Jen Chang

**Affiliations:** a Graduate Institute of Medical Sciences, College of Medicine, Taipei Medical University, Taipei, Taiwan; b School of Dentistry, College of Oral Medicine, Taipei Medical University, Taipei, Taiwan; c Department of Oral Medicine, Infection and Immunity, Harvard School of Dental Medicine, Boston, USA; d Division of Oral Rehabilitation and Center of Pediatric Dentistry, Department of Dentistry, Taipei Medical University Hospital, Taipei, Taiwan; e Department of Biomedical Engineering, Ming-Chuan University, Taoyuan, Taiwan; f Department of Anatomy and Cell Biology, School of Medicine, College of Medicine, Taipei Medical University, Taipei, Taiwan; g Dental Department, Taipei Medical University, Shuang-Ho Hospital, New Taipei City, Taiwan

**Keywords:** Periodontitis, Oral microbiota, Dental plaque, Full-length 16S rRNA sequencing, DADA2 bioinformatics pipeline

## Abstract

**Background/Purpose:**

Periodontitis is a chronic inflammatory disease that disrupts oral microbial homeostasis and contributes to systemic inflammation. While previous studies have focused on subgingival microbiota, the role of supragingival plaque as an early microbial reservoir remains underexplored. Most previous studies have relied on short-read 16S rRNA sequencing but limited detailed classification. This study uses Third-Generation Sequencing (TGS) with full-length 16S rRNA sequencing and DADA2-based error correction to better characterize the supragingival microbiome in periodontitis.

**Materials and methods:**

A total of 30 participants (15 periodontitis patients and 15 healthy controls) were recruited. Supragingival plaque samples were collected, and full-length 16S rRNA sequencing was performed using a TGS platform. Sequencing data were processed with DADA2 for error correction and taxonomic classification using the Human Oral Microbiome Database (HOMD), including diversity indices and Principal Component Analysis (PCA), which were conducted to compare microbial compositions between groups.

**Results:**

The periodontitis group exhibited significantly higher microbial diversity (*P* < 0.05) and enrichment of key periodontal pathogens, including *Porphyromonas*, *Prevotella*, *Fusobacterium*, and *Treponema*. On the other hand, commensal bacteria such as *Streptococcus* and *Neisseria* were more abundant in the healthy group. PCA demonstrated distinct clustering patterns, indicating supragingival microbial shifts associated with disease progression.

**Conclusion:**

This study provides the high-resolution microbial profiling of supragingival plaque in periodontitis using full-length 16S rRNA sequencing. The findings suggest that supragingival plaque may serve as an early-stage reservoir for periodontal pathogens, which leads to subgingival colonization and disease progression. It highlights the potential of microbial biomarkers in early diagnosis and suggests that targeted microbiome-based therapies could be developed to restore microbial balance in periodontal disease. This method offers greater precision in bacterial identification and future translational potential for personalized periodontal care.

## Introduction

Periodontal disease is a chronic inflammatory condition affecting the supporting structures of teeth and is one of the most prevalent oral diseases worldwide. Epidemiological studies estimate that 20–50 % of the global population is affected by periodontitis, with prevalence increasing with age. Based on the WHO's Community Periodontal Index of Treatment Needs (CPITN), periodontitis affects 49 % of elderly individuals worldwide, and the prevalence is even higher in Taiwan at 73 %.^1^ Periodontitis shift from a symbiotic to a dysbiotic oral biofilm triggers inflammation and progressive tissue destruction.^2^ The oral microbiota plays a key role in periodontal health and disease. The tooth surface allows microorganisms to form biofilms. As microbial dysbiosis develops, periodontal pathogens colonize the biofilm and spread into subgingival sites, leading to disease progression^3^ Studies indicate that patients with periodontitis exhibit higher microbial diversity than healthy individuals which suggests an altered bacterial ecosystem.^4^ 16S rRNA sequencing have enhanced our ability to analyze the oral microbiota. Understanding microbial shifts in periodontitis can provide insights into its pathogenesis and potential strategies for microbiome-targeted therapies.

The Human Oral Microbiome Database (HOMD) was expanded in 2017 to include both cultivable (70 %) and uncultivable (30 %) prokaryotic species, providing a more comprehensive reference for oral microbiome studies.^5^ Research has shown that *Prevotella* and *Veillonella* are more abundant in the saliva of periodontitis patients.^6^ In supragingival plaque, *Streptococcus* species and *Candida albicans* form structured biofilms, which later become colonized by periodontal pathogens.^7^^,^^8^ Periodontitis patients have higher levels of *Prevotella intermedia*, *Prevotella nigrescens*, *Fusobacterium nucleatum*, *Treponema forsythia*, and *Treponema denticola* than healthy individuals.^9^ Some periodontal pathogens are detected in supragingival plaque even before appearing in subgingival sites, suggesting that supragingival biofilms serve as reservoirs for infection. Comparative studies show that *Actinomyces* species are dominant in both healthy supragingival and subgingival plaque, while *Porphyromonas gingivalis*, *Tannerella forsythia*, and *Treponema denticola* are more abundant in periodontitis biofilms. The major difference between health and disease is the shift in orange-complex and red-complex bacteria.^10^^,^^11^ Traditional culturing methods were used to identify oral bacteria based on growth conditions, antibiotic susceptibility, and microscopy. However, many oral bacteria still remain unculturable.^12^^,^^13^ The development of next-generation sequencing (NGS) has revolutionized large-scale microbial profiling. More recently, TGS has further improved long-read genome sequencing and simplified genome assembly. DADA2 has also enhanced sequencing accuracy by removing errors and resolving unique sequence variants.^14^^,^^15^

The disruption of microbial homeostasis in periodontitis leads to a dysbiotic oral microbiome, which may contribute to systemic inflammation and disease progression.^16^ Despite growing evidence linking periodontitis to systemic health, the specific microbial differences between healthy and periodontitis-associated supragingival plaque remain insufficiently explored. Although previous studies have investigated the oral microbiome using 16S rRNA sequencing relied on short-read sequencing methods, which limit taxonomic resolution.^17^ To address these research gaps, this study carries to characterize the microbial composition of supragingival plaque in periodontitis and healthy individuals using Third-Generation Sequencing (TGS) technology with full-length 16S rRNA sequencing and DADA2-based error correction. A more detailed understanding of the microbial composition in periodontal health and disease could help identify key bacterial taxa linked to periodontitis progression and their potential systemic implications. We hypothesize that supragingival plaque harbors distinct microbial profiles between health and disease, with periodontitis patients exhibiting a higher abundance of known periodontal pathogens. Furthermore, we anticipate that full-length 16S rRNA sequencing will enhance bacterial taxonomic resolution, allowing for more precise microbial characterization than traditional short-read sequencing methods. This study also seeks to determine whether supragingival plaque is an early-stage reservoir for periodontal pathogens. It provides new perspectives on periodontal disease development and potential microbiome-targeted interventions.

This study investigated the microbial differences in supragingival plaque between periodontitis patients and healthy individuals using TGS technology with full-length 16S rRNA sequencing and DADA2-based error correction to provide a more precise characterization of the oral microbiome in periodontal health and disease by achieving higher taxonomic resolution. By integrating advanced sequencing technology, this study contributes to a deeper understanding of the complex microbial interactions in periodontal disease and their broader implications for oral and systemic health.

## Materials and methods

### Overall study design

This study was designed to compare the supragingival plaque microbiota between healthy individuals and periodontitis patients using full-length 16S rRNA gene sequencing. A total of 30 participants (15 periodontitis patients and 15 healthy controls) were recruited from a clinical setting, with demographic data and periodontal parameters recorded. All oral examinations and supragingival plaque sample collections were conducted by the same operator, who was trained at the periodontal department of Taipei Medical University (Taipei, Taiwan), ensuring consistency and reliability in clinical assessments. Supragingival plaque samples were collected, followed by DNA extraction, library preparation, and sequencing using Third-Generation Sequencing (TGS) technology. Microbial composition and diversity were analyzed using bioinformatics pipelines, and statistical methods were applied to evaluate differences in microbial community structure. Clinical and microbiome data were integrated to explore microbial shifts associated with periodontitis.

### Participants recruitment

This study was approved by the Institutional Review Board (IRB) of Taipei Medical University (Approval No: TMU-JIRB 20200317). Participants were recruited from Shuang Ho Hospital, New Taipei City, Taiwan, following a structured selection process based on predefined inclusion and exclusion criteria, as shown in Table 1. Eligible participants were screened during routine dental visits, and those who met the criteria were invited to participate. A total of 30 subjects were enrolled, comprising 15 healthy individuals in the control group and 15 patients diagnosed with periodontitis. Informed consent was obtained from all participants before their inclusion in the study. Based on clinical evaluation, all periodontitis subjects met the diagnostic criteria for stage IV periodontitis, which is defined by the 2017 World Workshop classification as characterized by severe attachment loss, deep probing depth, and functional compromise.

**Table 1:** Demographic characteristics of subjects in the study.

Basic profile	Control	Periodontitis
Sample size, *N*	15	15
Male: Female	9:6	6:9
Age, years	43 (31–61)	54 (48–64)

To ensure consistency and reliability in subject selection, periodontitis was diagnosed by experienced periodontists based on clinical parameters, including probing depth (PD), clinical attachment loss (CAL), and bleeding on probing (BOP), in accordance with the classification guidelines of the American Academy of Periodontology (AAP). Healthy individuals had no clinical signs of periodontitis or gingival inflammation. Participants with systemic conditions known to affect periodontal health, such as diabetes, immunodeficiency disorders, or recent antibiotic use, were excluded from the study.

### Demographic data record and oral examination

**Figure 1:**
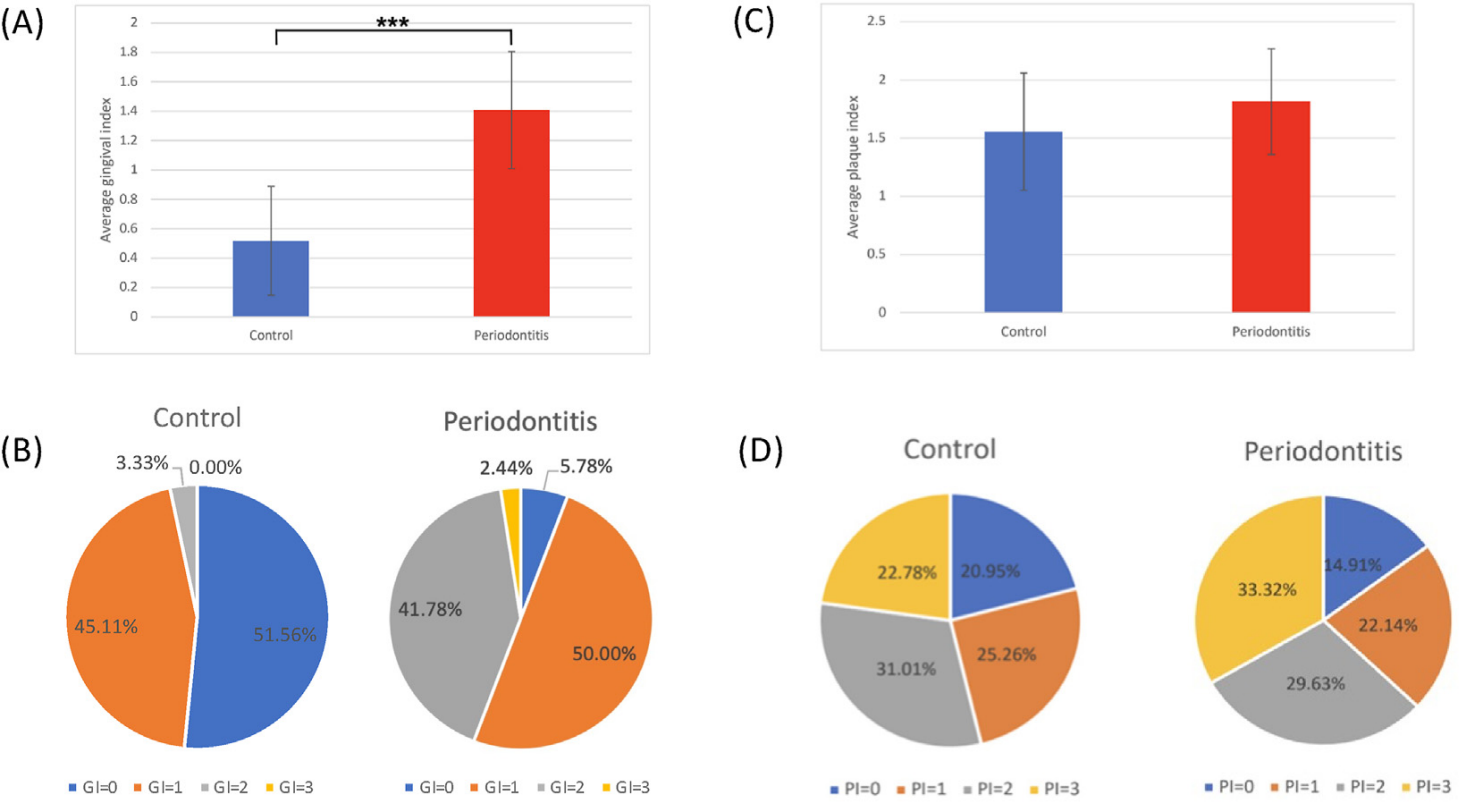
Average values **(A)** and percentages **(B)** of gingival index (GI), as well as average values **(C)** and percentages **(D)** of plaque index (PI) for control and periodontitis groups. (∗∗∗*P* < 0.001 analyzed using T-test).

**Figure 2:**
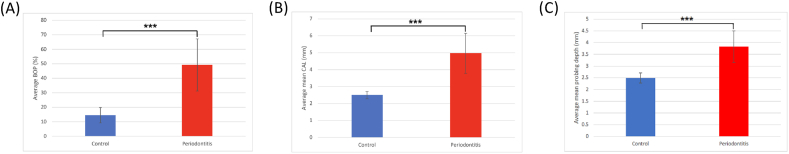
**(A)** Average bleeding on probing (BOP) **(B)** clinical attachment loss (CAL)**,** and **(C)** probing depth **for** control and periodontitis groups. (∗∗∗*P <* 0.001 analyzed using T-test).

**Figure 3:**
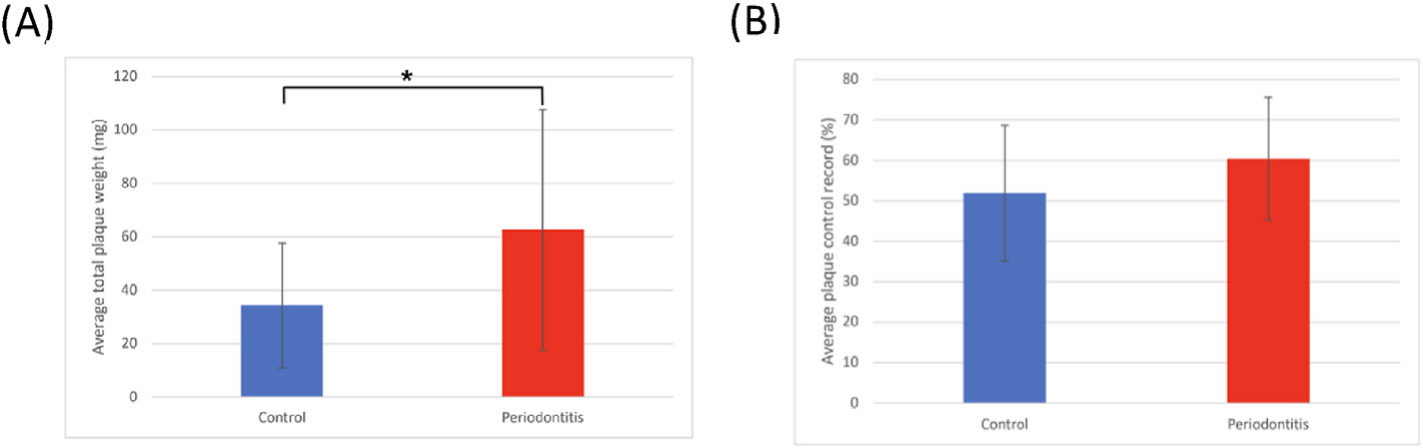
**(A)** Average total dental plaque weight, and **(B)** Plaque control record. (∗*P <* 0.05 analyzed using T-test).

Before participating in the study, all subjects were provided with a detailed explanation of the research. Written informed consent was obtained from each participant prior to data collection. Demographic information, including gender, age, systemic health conditions, smoking status, and alcohol consumption habits, was documented through a structured questionnaire. A trained clinician conducted oral examinations under proper lighting in a dental chair. Clinical parameters were recorded to assess oral and periodontal health. These included the Decayed, Missing, and Filled Teeth (DMFT) score, plaque control record, plaque index (PI), and comprehensive periodontal charting. The periodontal assessment included probing depth (PD), gingival recession, furcation involvement, tooth mobility, bleeding on probing (BOP), and gingival index (GI). All measurements were performed using standardized periodontal probes and diagnostic instruments to ensure consistency and reliability. The specific methods and instruments used for each clinical parameter are detailed in Table 2 and Figure 1, Figure 2, Figure 3.

**Table 2:** Decayed, missing, and filled teeth scores of subjects.

Dental profile	Control	Periodontitis
Decayed, missing, and filled teeth (DMFT) score	13.0 ± 4.6	14.2 ± 6.3
Number of decayed teeth (DT value)	1.3 ± 2.3	0.7 ± 1.6
Number of missing teeth (MT value)	4.1 ± 2.3	6.1 ± 3.5
Number of filled teeth (FT value)	8.3 ± 5.6	7.4 ± 4.1

### Microbiome library construction and sequencing

**Figure 4:**
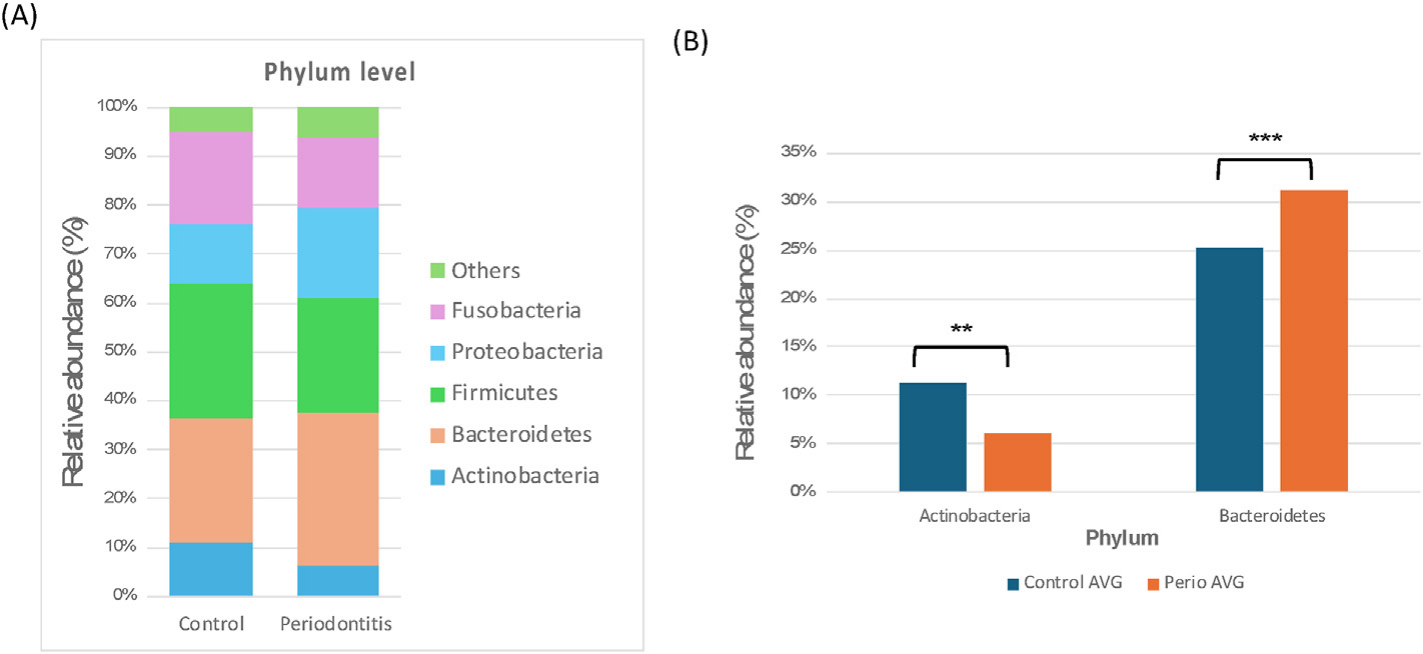
**(A)** Relative abundance of the top six bacterial taxa at the phylum level presented as a stacked bar chart, and **(B)** statistical differences in bacterial relative abundance.

**Figure 5:**
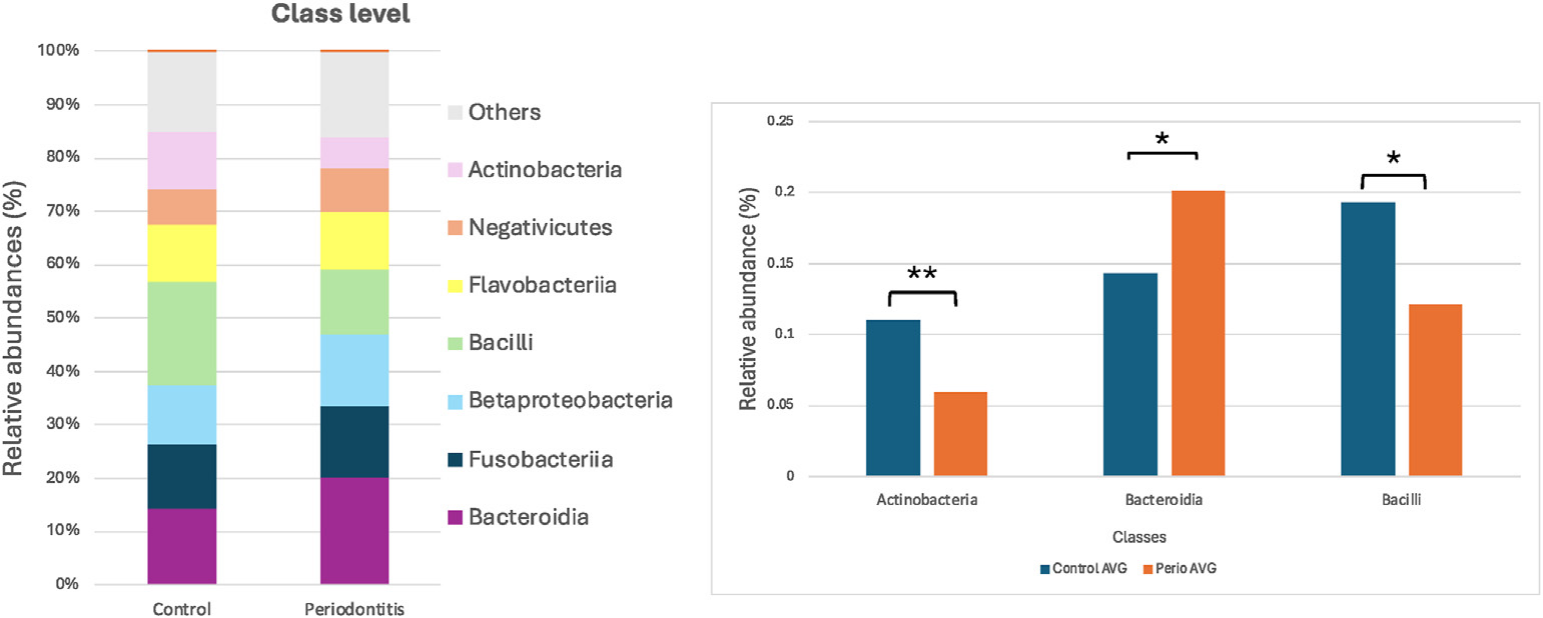
**(A)** Relative abundance of the top seven bacterial taxa at the class level is presented as a stacked bar chart, and **(B)** statistical differences in bacterial relative abundance.

**Figure 6:**
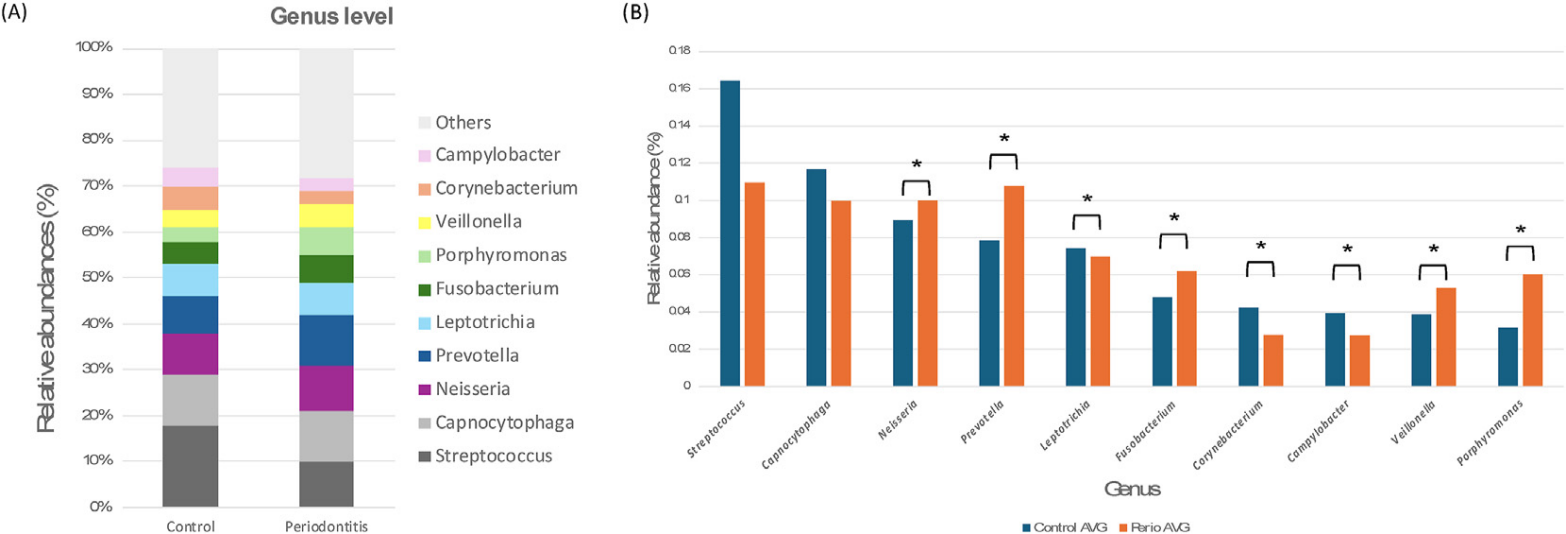
**(A)** Relative abundance of the top 10 bacterial taxa at the genus level **(B)** Comparison of the relative abundance of the 10 genera between control and periodontitis groups. (∗*P <* 0.05 analyzed using T-test).

Microbial analysis was conducted using full-length 16S rRNA gene sequencing based on Third-Generation Sequencing (TGS) technology to achieve high-resolution taxonomic profiling of supragingival plaque microbiota. This approach provides a more comprehensive assessment of microbial diversity compared to conventional short-read sequencing methods. First, total bacterial DNA was extracted from supragingival plaque samples using a standardized extraction protocol to ensure high-quality template DNA for sequencing. The extracted DNA underwent polymerase chain reaction (PCR) amplification of the entire 16S rRNA gene, followed by rigorous quality control (QC) assessment to confirm DNA integrity and purity. Only samples that met the QC criteria were used for library preparation, where DNA fragments were processed into sequencing-ready libraries. Once library construction was completed, high-throughput full-length 16S rRNA gene sequencing was performed using a TGS platform, enabling accurate taxonomic classification at the species level. Following sequencing, raw data processing and quality filtering were conducted, and sequencing errors were corrected using the Divisive Amplicon Denoising Algorithm 2 (DADA2). The high-quality sequences were then aligned against the Human Oral Microbiome Database (HOMD) to achieve precise bacterial community profiling. This analytical workflow enabled a comprehensive characterization of the supragingival microbiota at full-length resolution, providing deeper insights into microbial composition and structure. A schematic overview of the sequencing pipeline is presented from Figure 4, Figure 5, Figure 6.

### Statistical analysis

All statistical analyses were performed using IBM SPSS Statistics (version 22, IBM Corp., Armonk, NY, USA). To compare clinical parameters between the control and periodontitis groups, a Student's t-test was used for normally distributed data. For microbial composition analysis, the Kruskal–Wallis test was applied to assess differences across multiple groups, while the Wilcoxon rank-sum test was used for pairwise comparisons where appropriate. A significance level of *P* < 0.05 was considered statistically significant.

## Results

### Demographic data and dental information

The demographic characteristics of the control and periodontitis groups are presented in Table 1. Each group consists of 15 subjects. The age range of the control group is 31–61 years, with a median age of 43 years, while the periodontitis group has an age range of 48–64 years and a median age of 54 years.

The dental information, including the Decayed, Missing, and Filled Teeth (DMFT) score, is presented in Table 2. The DMFT score was 13.0 ± 4.6 in the control group and 14.2 ± 6.3 in the periodontitis group. However, there is no statistical significance in the DMFT score, number of decayed teeth, number of missing teeth, or number of filled teeth between the control and periodontitis groups. These results suggest that the overall dental caries experience does not differ between the groups.

#### Gingival index (GI) and pPlaque index (PI)

The periodontitis group exhibited a significantly higher average gingival index (GI) of 1.41 compared to 0.52 in the control group (*P* < 0.001, Fig. 1A). Regarding the distribution of GI scores, the control group had a significantly higher proportion of subjects with GI = 0 (51.56 %) compared to 5.78 % in the periodontitis group (*P* < 0.001). Conversely, the periodontitis group showed a significantly higher proportion of subjects with GI = 2 (41.78 %) compared to 3.33 % in the control group (*P* < 0.001, Fig. 1B).

For the plaque index (PI), the periodontitis group showed a slightly higher average PI (1.81) compared to the control group (1.55), but this difference was not statistically significant (Fig. 1C). Regarding PI distribution percentage, the control group had slightly higher percentages of PI = 0, PI = 1, and PI = 2, while the periodontitis group showed a higher percentage of PI = 3 (Fig. 1D).

### Periodontal health parameters and plaque accumulation

The periodontitis group demonstrated significantly worse periodontal health compared to the control group across all measured parameters. The average bleeding on probing (BOP) was 49.16 % in the periodontitis group, significantly higher than 14.52 % in the control group (*P* < 0.001, Fig. 2A). Similarly, in terms of the clinical attachment loss (CAL) was significantly greater in the periodontitis group, with an average of 4.97 mm compared to 2.49 mm in the control group (*P* < 0.001, Fig. 2B). Moreover, the average of mean probing depth (PD) was also significantly increased in the periodontitis group (3.83 mm) compared to the control group (2.49 mm, *P* < 0.001), as shown in Fig. 2C.

The periodontitis group demonstrated a significantly higher average of total plaque weight (62.63 mg), which is significantly higher than control group (34.27 mg, *P* < 0.05, Fig. 3A). When considering the average plaque weight per tooth, a similar pattern could be observed, in which the periodontitis group exhibited a statistically significantly higher figure (2.4_46 mg) than that of the control group (1.22 mg) (*P* < 0.05) (data not shown). However, in terms of plaque control record, the periodontitis group showed a slightly higher average plaque control record (60.45 %) compared to the control group (51.88 %). However, this difference was not statistically significant (Fig. 3B).

### Microbiota composition at the phylum, class and genus level

The oral microbiota composition exhibited differences between the control and periodontitis groups, as shown in Fig. 4A. The top six most abundant phyla were *Actinobacteria*, *Bacteroidetes*, *Firmicutes*, *Fusobacteria*, *Proteobacteria*, and others, which exhibited notable variations in relative abundance. In particular, as illustrated in Fig. 4B, the relative abundance of *Actinobacteria* was significantly lower in the periodontitis group compared to the control group (*P* < 0.01). In contrast, *Bacteroidetes* were significantly more abundant in the periodontitis group (*P* < 0.001). These findings indicate that periodontal disease is associated with a dysbiotic shift in the oral microbiota characterized by reduced commensal bacteria and increased periodontal pathogens.

At the class level, analysis of the oral microbiota revealed notable differences between the control and periodontitis groups. The top seven most abundant classes identified were *Actinobacteria*, *Bacteroidia*, *Clostridia*, *Fusobacteriia*, *Gammaproteobacteria*, *Bacilli*, and others, with distinct variations in their relative abundances, as shown in Fig. 5A. The statistical comparisons indicated that the relative abundance of *Actinobacteria* was significantly lower in the periodontitis group compared to the control group (*P* < 0.01). In contrast, *Bacteroidia* were significantly more abundant in the periodontitis group (*P* < 0.05). Additionally, *Bacilli* exhibited a significant decrease in the periodontitis group compared to the control group (*P* < 0.05) (Fig. 5B). These findings suggest that periodontal disease is associated with a shift in the microbial community structure at the class level, characterized by a reduction in commensal bacteria such as *Actinobacteria* and *Bacilli*, along with an increase in potential periodontal pathogens such as *Bacteroidia*.

Analysis of the oral microbiota at the genus level revealed significant differences between the control and periodontitis groups. Fig. 6A illustrates the relative abundance of the top 10 bacterial genera. For statistical analysis (Fig. 6B), it was observed that the periodontitis group exhibited a significant increase in the relative abundance of *Porphyromonas* (*P* < 0.05), *Prevotella* (*P* < 0.05), *Fusobacterium* (*P* < 0.05), and *Veillonella* (*P* < 0.05) compared to the control group. Additionally, *Neisseria* showed a significant increase in the periodontitis group (*P* < 0.05), suggesting its potential role in periodontal disease progression.^18^ Conversely, the relative abundance of *Streptococcus*, *Leptotrichia*, and *Corynebacterium* was significantly lower in the periodontitis group (*P* < 0.05). These findings suggest a shift in the oral microbial community associated with periodontal disease, which is characterized by an increase in periodontal pathogens such as *Porphyromonas*, *Prevotella*, and *Fusobacterium*, along with a decrease in commensal bacteria like *Streptococcus* and *Capnocytophaga*.

### Alpha diversity and PCA-based beta diversity analysis

**Figure 7:**
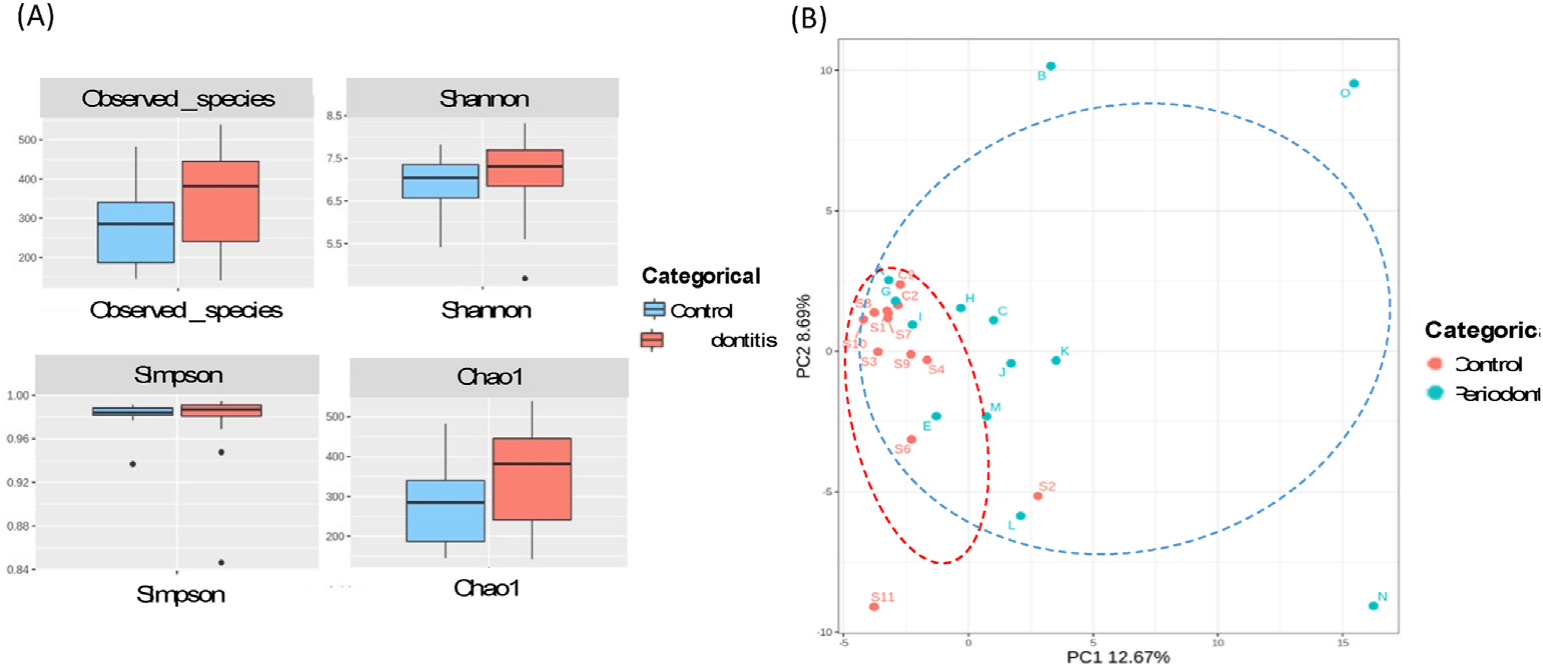
**(A)** Alpha diversity indices, including observed species, Shannon index, Simpson index, and Chao1 index for the control and periodontitis groups. **(B)** Principal component analysis (PCA) plot illustrating the separation between control and periodontitis groups based on microbial composition.

Alpha diversity analysis revealed differences in microbial richness and evenness between the control and periodontitis groups. As shown in Fig. 7A, the periodontitis group exhibited higher observed species count, Shannon index, and Chao1 index compared to the control group, suggesting increased microbial diversity in the diseased state. Besides, the Simpson index also showed a slightly higher median value in the periodontitis group, indicating a potential shift in microbial dominance. Regarding the findings of observed species, individuals with periodontitis harbor a more diverse and complex microbiota than healthy individuals, potentially contributing to disease progression. Subsequently, beta diversity analysis using plot of Principal Component Analysis (PCA) demonstrated a distinct clustering pattern like a coordinate diagram after ordination between the control and periodontitis groups (Fig. 7B). The control group exhibited a more compact clustering pattern, whereas the periodontitis group showed a broader distribution. This broader distribution suggests greater inter-individual variability in the periodontitis-associated microbiota, reflecting a more heterogeneous microbial community.

## Discussion

Periodontitis is clinically characterized by increased gingival inflammation, plaque accumulation, and periodontal attachment loss from our collection data. As shown in Fig. 1, the periodontitis group exhibited significantly higher GI and PI compared to the control group (*P* < 0.001), reflecting increased gingival inflammation and biofilm accumulation.^19^ Increased plaque accumulation may create a favorable niche for anaerobic pathogens and drives further inflammation and tissue destruction.^20^ Compared to the plaque control record and plaque index, the plaque weight may reflect clinically more on the periodontal status of the subjects. Similarly, the BOP, CAL and PD were significantly elevated in the periodontitis group from Fig. 2 (*P* < 0.001). BOP is a primary indicator of active inflammation, which increase in the periodontitis group suggests a heightened inflammatory response to bacterial biofilms. The consensus report of the 2017 World Workshop^2^ on the classification of periodontal and peri-implant diseases and conditions in which periodontitis is featured by the continuous breakdown of periodontal supporting tissues and manifests clinically as increased probing depth, clinical attachment loss, and gingival inflammation. The accumulation of bacterial plaque concomitantly with dysbiosis initiates and deteriorates periodontitis, while the clinical attachment loss resulting from periodontitis provides more supragingival surfaces for bacteria to adhere. The substantial increase in CAL and PD also reflects periodontal tissue destruction and alveolar bone resorption, which are consistent with the progressive nature of periodontitis.^21^^,^^22^ Furthermore, the periodontitis group exhibited a significantly higher total dental plaque weight (*P* < 0.05, Fig. 3A). A statistically significant difference in the plaque weight in which periodontitis had 1.8 times as much average total plaque weight as the control group and 2 times as much average plaque weight per tooth as the control group. It indicates plaque accumulation may be a key driver of periodontal disease.

Microbial dysbiosis in periodontitis manifests as alterations at phylogenetic levels, which can be detected by third generation sequencing method. At the phylum level, we observed significant changes in the microbial composition between the control and periodontitis groups (Fig. 4). The periodontitis group exhibited a significant decrease in *Actinobacteria* and an increase in *Bacteroidetes* (*P* < 0.001). This trend is consistent with previous research.^23^ *Actinobacteria* is commonly associated with oral health and play a critical role in maintaining microbial homeostasis, whereas *Bacteroidetes* are known for anaerobic metabolism and inflammation-driven dysbiosis. In phylum-level shift indicates that periodontitis is characterized by an imbalance favoring inflammatory bacterial composition. Further analysis at the class level from Fig. 5 revealed significant differences in microbial composition. Actinobacteria were significantly depleted in the periodontitis group (*P* < 0.01), while *Bacteroidia* and Bacilli showed contrasting trends. The enrichment of *Bacteroidia* is notable because it contains well-documented periodontopathogens such as *Porphyromonas* and *Prevotella*.*^24^* On the contrary, Bacilli is a class that includes several health-associated species, including *Streptococcus*, which was significantly reduced in the periodontitis group (*P* < 0.05). This suggests that the depletion of protective *Streptococcus* species may allow pathogenic species to dominate, further supporting the concept of microbial dysbiosis as a driver of periodontal disease.^25^^,^^26^

At the genus level, Fig. 6 revealed significant shifts in key bacterial genera associated with periodontitis. The results are in accordance with a study by Ximénez^10^ stating that the main differences between health and periodontitis were in the levels of “orange” and “red” complex species, which are associated with periodontal inflammation. Notably, the periodontitis group exhibited a significant increase in *Porphyromonas*, *Prevotella*, *Fusobacterium*, and *Veillonella*, all of which are well-documented periodontal pathogens known for their role in biofilm formation, immune evasion, and tissue destruction.^27^^,^^28^ These periodontal bacteria release virulence factors and inhibit the bactericidal activity of leucocytes, resulting in the host's inflammatory immune response and leading to clinical attachment loss. Particularly, *porphyromonas gingivalis* has been recognized as a keystone pathogen that manipulates host immune responses and promotes microbial dysbiosis.^29^^,^^30^ Additionally, *Prevotella intermedia* often coexists with *P. gingivalis* under anaerobic conditions, which provide each other with essential growth factors and collectively exacerbate local inflammation. Likewise, *Fusobacterium nucleatum* is also known as a bridging organism which facilitates the coaggregation of early and late colonizers, thereby stabilizing the subgingival biofilm.^31^^,^^32^ There is also evidence suggesting that while *Veillonella parvula* is generally considered a commensal species. Under certain conditions, interact with other pathogens and intensify inflammation in oral cavity^33^^,^^34^ Conversely, a significant reduction in *Streptococcus*, *Leptotrichia*, and *Corynebacterium* was observed in the periodontitis group, which indicated a loss of beneficial commensal bacteria that contribute to oral health. For example, *Streptococcus sanguinis* from *Streptococcus* species can produce hydrogen peroxide and bacteriocins to inhibit periodontal pathogens.^35^^,^^36^ However, its decreased abundance in the periodontal disease group may result in the dominance of pathogenic periodontal bacteria.

Alpha diversity from Fig. 7 A provides the insights into the microbial richness and evenness from the dental plaque. In our study, periodontitis group demonstrates greater species richness and evenness along with higher Alpha diversity than the control group, which corresponds with previous studies.^10^^,^^37^ Periodontitis group exhibited significantly higher observed species count, Shannon index, and Chao1 index, indicating an increase in microbial richness compared to the control group. These findings suggest that periodontal disease is associated with an expansion of bacterial taxa because of the overgrowth of pathogens in response to an inflammatory microenvironment. Interestingly, Simpson's index was also slightly higher in the periodontitis group. The higher Simpson's index suggests that while microbial richness increased. The shift in microbial diversity highlights the complex interplay between host immune responses and microbial adaptation in periodontitis progression. Similarly, Beta diversity analysis evaluates differences in microbial composition distribution between two groups. It offers insights into how distinct the microbial communities are regarding health and disease. As shown in Fig. 7B, the healthy group showed greater sample similarity or higher beta diversity. PCA demonstrated a clear separation, indicating substantial differences in microbial community composition. A possible model of the microbial shift from healthy to periodontitis may be a distinct increase in the diversity of supragingival bacteria with greater heterogenicity between periodontally-challenged subjects. This clustering suggests that periodontitis is associated with a dysbiotic microbial ecosystem.^38^^,^^39^

Although our findings could distinguish periodontitis, this study still has several limitations. First, the sample size was relatively small, which may limit the generalizability of the results to a broader population. Second, this study employed 16S rRNA gene sequencing, which provides taxonomic classification but lacks functional insights into microbial activity. While 16S sequencing is valuable for characterizing microbial composition, it does not capture metagenomic or transcriptomic changes that may be crucial in periodontal disease pathogenesis. Third, as a cross-sectional study, our research cannot establish causality between microbial shifts and periodontal disease progression. The observed dysbiotic microbial profile may be either a cause or a consequence of periodontitis. Lastly, this study did not extensively control environmental and behavioral factors such as smoking, oral hygiene practices, and systemic diseases. These factors may influence microbial composition and should be carefully considered in future research. Future research should integrate multi-omics approaches to better understand the complex interplay between the host immune response and the oral microbiome, ultimately guiding the development of personalized microbiome-targeted therapies for periodontal disease management. In addition, machine learning algorithms such as Random Forest or Support Vector Machines could be applied to the microbial data to build predictive models for periodontitis. This approach may help identify microbial biomarkers and support early diagnosis or precision treatment.

These findings provide key insights into the microbial shifts associated with periodontitis. Healthy individuals tend to harbor a more uniform supragingival microbiota, whereas those with periodontitis exhibit increased microbial diversity and inter-individual heterogeneity. This suggests that as periodontal disease progresses, the supragingival microbiota undergoes a distinct transformation, characterized by greater bacterial diversity and variability among individuals. Understanding these shifts may offer valuable perspectives for developing microbiome-based diagnostic and therapeutic strategies for periodontal disease.

This study demonstrates that periodontitis is associated with distinct microbial shifts in the supragingival plaque, as revealed by full-length 16S rRNA sequencing. The periodontitis group exhibited significantly higher microbial diversity (*P* < 0.05). The significant enrichment of *Prevotella*, *Fusobacterium*, and *Porphyromonas* taxa was commonly implicated in inflammation and tissue destruction, further emphasizing their role as key periodontal pathogens. Conversely, the marked depletion of *Streptococcus* and *Neisseria* were dominant commensal genera in healthy individuals. This microbial imbalance may facilitate the overgrowth of pathogenic species and contribute to disease progression. Furthermore, supragingival plaque may serve as an early-stage reservoir for periodontal pathogens, potentially contributing to disease progression before subgingival colonization. The use of full-length 16S rRNA sequencing provided higher taxonomic resolution, enabling more precise identification of periodontitis-associated taxa compared to conventional short-read sequencing. These compositional patterns may hold diagnostic potential. A shift in supragingival microbiota, characterized by increased periodontopathogens and reduced protective taxa, could be an early signal for disease onset, informing timely preventive or therapeutic interventions. Identifying supragingival microbial biomarkers could facilitate early detection strategies and microbiome-targeted interventions to restore microbial balance and improve periodontal health.

## Declaration of competing interest

The authors have declared that there are no competing interests.
